# Causes and Effects of Postoperative Sleep Disorders and Treatment Strategies for Preoperative, Intraoperative, and Postoperative Settings—A Narrative Review

**DOI:** 10.3390/clockssleep7030029

**Published:** 2025-06-22

**Authors:** Michael J. Nelson, Darien A. Yu, Augustine V. H. Ha, Mark R. Wakefield, Yujiang Fang

**Affiliations:** 1 Department of Microbiology, Immunology & Pathology, Des Moines University College of Osteopathic Medicine, West Des Moines, IA 50266, USA; michael.j.nelson@dmu.edu; 2 Department of Surgery, University of Missouri School of Medicine, Columbia, MO 65212, USA; dayqdd@missouri.edu (D.A.Y.); avhmy8@missouri.edu (A.V.H.H.);; 3 Ellis Fischel Cancer Center, University of Missouri School of Medicine, Columbia, MO 65212, USA

**Keywords:** postoperative sleep disturbances, delayed recovery, surgery, contributing factors, consequences, interventions, treatments

## Abstract

Sleep is an essential part of everyday life and disturbed sleep can produce numerous deleterious effects on the body. An especially prevalent and detrimental subset of sleep disturbances are sleep disturbances that occur in patients during the postoperative period. To better understand these disorders and how to treat them, a literature search was conducted to identify and consolidate recent advancements in this field. This narrative review discusses the structure of a typical night of sleep and the causes, effects, and treatment strategies of Postoperative Sleep Disturbances (PSDs). Factors that contribute to the development of PSDs have been identified at all stages of the surgical process, including the preoperative, intraoperative, and postoperative timepoints. Exposure to these factors can accumulate during each step and may decrease the quality of sleep postoperatively. The development of PSDs has been found to affect multiple systems throughout the body and can ultimately lead to poorer recovery times and increased postoperative mortality. As a result, multiple therapeutic approaches are being investigated for their role in reducing the prevalence of these disorders. This has revealed promising interventions throughout the surgical process, such as preoperative nerve blocks, intraoperative infusions, and postoperative behavioral interventions. However, despite these successful findings, work still needs to be completed to optimize these techniques and generalize intervention strategies.

## 1. Introduction

Sleep is an essential part of life for most organisms [1]. Humans spend a significant portion of most days asleep, and although the amount of sleep that our bodies require changes as we age, the basic need for sleep remains consistent. Insufficient sleep can lead to sensory impairments, decreased cognitive performance, and homeostatic dysfunctions [1]. These consequences affect the healing process, making sleep an especially important factor during the postoperative period.

Given this importance, a large amount of research has recently been devoted to preventing and treating postoperative sleep disturbances (PDSs). Due to this increase in publications, we felt that many of the current review articles on this topic were either slightly outdated or lacked a broad discussion on treatment strategies at all points of the surgical process, including pharmaceutical and non-pharmaceutical interventions. As a result, a literature review was conducted by searching though several databases (primarily PubMed) to identify recent publications on PSDs. Priority was given to articles that were published within the last 5–10 years and then assessed for relevance. The aim of this narrative review is to provide an updated analysis on the causes, effects, and treatment strategies of PSDs, with emphasis on preoperative, intraoperative, and postoperative interventions.

### 1.1. Background of Sleep

To understand sleep’s role in daily life and recovery, it is important to have a basic understanding of how sleep is structured. The feeling of sleepiness occurs due to a combination of circadian influences and the accumulation of wastes throughout the day, which triggers a cascade of neurological changes that lead to sleep [1,2]. Sleep itself is broken into two major categories, Non-Rapid Eye Movement (NREM) and Rapid Eye Movement (REM) sleep. NREM sleep is further broken into N1 (the very first stage of sleep) followed by N2 and N3 sleep, with each stage representing deeper sleep and slower EEG waves [3]. Throughout the night, NREM sleep gives way to REM sleep, during which the brain enters a wakefulness-like state as dreams become more prevalent and organized [4,5]. A typical night of sleep will consist of multiple cycles that progress as follows: N1 → N2 → N3 → N2 → REM [6,7]. This combination of REM and NREM sleep each night is thought to play an important role in the removal of neurological wastes, hormone regulation, memory consolidation, and information processing [4,5].

### 1.2. Sleep Monitoring Methods

Multiple techniques have been developed that allow researchers and doctors to better assess the structure of sleep and identify when it goes wrong. One of the main measurements used to assess sleep is sleep quality, which is based on a combination of quantitative measurements (e.g., sleep deprivation, sleep latency, and number of arousals) and subjective assessments (e.g., depth of sleep and restfulness) [8]. Because of sleep quality’s complex parameters, different techniques have been created to make more accurate methods of assessing it.

One of the main methods to analyze the objective portion of sleep quality is polysomnography. This technique simultaneously gathers data on neurophysiologic, cardiopulmonary, and other physiologic values over a period of several hours while a patient sleeps, providing both a quantitative and qualitative evaluation of sleep [3]. There are 4 different levels of polysomnographic studies that can be completed, with the highest level gathering measurements on EEG, EOG, EMG, ECG, airflow, O2 saturation, and respiratory effort. The large amount of data that this technique offers has made it an essential tool in diagnosing a variety of sleep disorders, including sleep-related breathing disorders, narcolepsy, parasomnias, and sleep-related seizure disorders [3]. More recently, portable polysomnography devices have been created that offer less complete monitoring but can be used at a patient’s own home, offering a more practical option for gathering data [3]. Outside of polysomnography, other objective measures of sleep used in research include actigraphy and consumer sleep trackers [9,10,11].

To analyze the subjective portion of sleep quality, multiple sleep questionnaires have been developed. One of the most popular is the Pittsburgh Sleep Quality Index (PSQI), which is a self-reported assessment given to patients on two occasions one month apart from each other [12,13,14]. It consists of 19 self-rated questions and gives a global score rated from 0 to 21 and 7 component scores rated from 0 to 3. Higher scores signal worse sleep quality and can differentiate poor sleepers from good sleepers [15]. This information is used by clinicians as an easy and subjective scale to study insomnia symptoms in patients [8]. Aside from the PSQI, many other questionnaires have been created, including the Jenkins Sleep Problem Scale, St. Mary’s Hospital Sleep Questionnaire, Karolinska Sleepiness Scale, Athens Insomnia Scale, and Richards–Campbell Sleep Questionnaire [14,16,17,18].

It should be noted, however, that, despite having multiple methods to analyze sleep both subjectively and objectively, a standardized way of assessing sleep disturbances in hospitalized patients does not exist. Instead, hospitals rely on a combination of history taking, nursing assessments, sleep studies, and questionnaires to evaluate a patient for sleep disturbances [19].

### 1.3. Benefits of Sleep and Issues with Not Sleeping Enough

As people mature, the amount of sleep they need gradually decreases up until early adulthood, when sleep requirements for an average person stabilize at 7–9 h of sleep a night [4]. While spending this amount of time each day in an incapacitated state is not ideal, it serves an important role by allowing the body to remove waste products that build up throughout the day and letting the brain consolidate and process memories [6]. When sleep is disrupted, whether through conscious decisions to avoid sleep or impaired sleep quality due to an underlying pathology, it affects multiple systems within the body. Sleep deprivation has been associated with altered autonomic nervous system function and respiratory control. Sleep also regulates hormone release in the body and sleep deprivation can cause serious dysfunction in the endocrine systems [1]. It also results in lower cognitive function and changes the body’s inflammatory response. If sleep disruption continues for long enough, the body will begin to force involuntary microsleeps to maintain proper homeostasis [4]. A specific source of sleep deprivation that has recently garnered a lot of research focus is sleep disturbances that occur postoperatively. This is an especially troublesome source of sleep disruption as many consequences of sleep deprivation can affect a patient’s ability to recover from the stress and trauma of surgery.

### 1.4. Postoperative Sleep Disturbances (PSDs)

PSDs are characterized as the abnormal quality and quantity of sleep or an altered sleep/wake rhythm that is experienced by patients following a surgical operation. They have been estimated to occur in 15–72% of patients following surgery, although that value can vary based on the type of surgery being performed and the postoperative environment the patients find themselves in [12,20]. One study found that 60% of patients who ended up in the ICU following surgery experienced PSD compared to only 25% of patients at home who experienced PSD [21]. In patients who do experience PSD, insomnia is one of the most common manifestations [22]. However, patients may also experience hypersomnia, decreased sleep time, increased awakening, and frequent nightmares which serve to disrupt the normal circadian rhythm and produce abnormal sleep architecture [18,22,23]. The polysomnographic testing of these patients reveals sleep fragmentation, a decreased amount (or even loss) of slow wave sleep, and reduced amounts of REM sleep [7,24,25].

These sleep disturbances are typically worse on the first night following surgery and gradually improve with time, but can be detectable for weeks afterwards [4,14]. One study found that 60% of patients experienced continued sleep disturbances 6 months after their operation [26]. Another found that 69% of patients who underwent arthroscopic rotator cuff repairs had disrupted sleep at 12 weeks post op [23]. The variation in the prevalence and persistence of PSD combined with the wide range of potential manifestations are part of the reason why PSD has received so much attention in recent years.

## 2. Etiology of PSD

**Figure 1:**
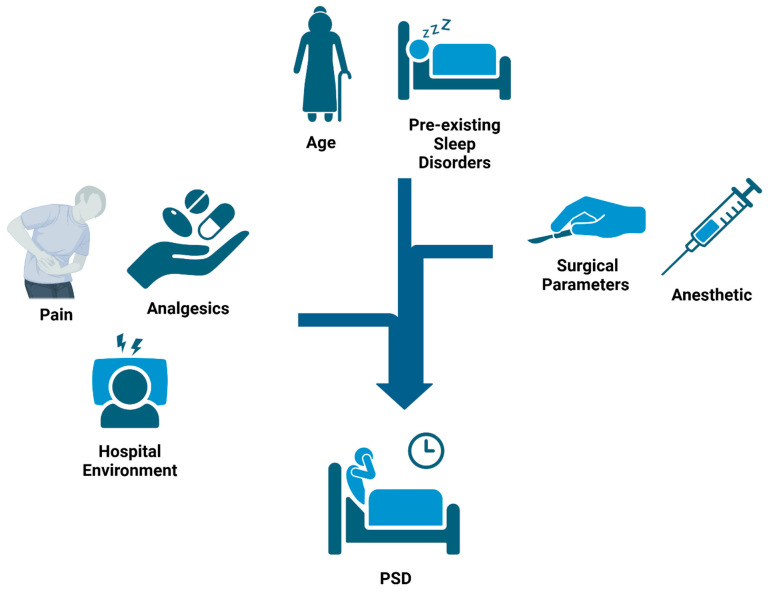
Causes of Postoperartive Sleep Disturbances (PSDs): Surgical patients are exposed to multiple factors throughout their hospital stay that contribute to the development of PSDs. Preoperatively, pre-existing sleep disorders and age play a large role in determining risk. Intraoperatively, factors such as surgical parameters and anesthetic agents can further alter the risk. Major factors in the postoperative period include surgical pain, analgesics, and the hospital environment. Exposure to these factors across the surgical timeline aggregate in a cumulative manner to affect a patient’s risk of developing PSDs.

The perioperative period is a stressful time for the patient. As shown in Figure 1, the process of preparing for an operation, the effects of the operation itself, and the postoperative care provided each expose a patient to stressors that may increase or decrease the appearance of PSDs [7]. Isolating and eliminating factors that directly affect the amount of stress a patient experiences in the perioperative period, without sacrificing the quality of care provided, is an important consideration when making treatment decisions. If these factors are managed poorly, patients can experience poor sleep months after the operation [27].

### 2.1. Nonmodifiable Factors

Like other complex conditions, some of the variables that predispose a patient to PSDs extend beyond a clinician’s realm of control. One such factor is the age of the patient. Age has a positive correlation with postoperative insomnia, partially due to age-related changes in drug sensitivity and toxicity [18,21,28]. In addition to this, endogenous melatonin production has been shown to decrease in patients over 55, which may further decrease sleep quality in this population [12]. Along with age, pre-existing sleep abnormalities, such as obstructive sleep apnea, hypoventilation, parasomnias, and insomnia, may cause or exacerbate the prevalence of PSD in certain patients [4]. While these factors may lie outside a clinician’s realm of control, it is important to take them into consideration when developing a care plan for each patient.

### 2.2. Hospital Environment

Removing immutable patient demographics from the equation, the hospital environment is one of the key areas of focus in treating PSDs. The hospital environment is defined as the sounds, lights, staff interactions, interactions with other patients, and other stimuli that the patient experiences (conscious or unconscious) while not in the operative room [4,21]. From their admittance pre-op until their discharge from the hospital, patients are almost continuously exposed to this environment, which has made it a point of emphasis for decreasing rates of PSDs. Research has shown that patients who were hospitalized for more than one week experienced a decrease in sleep quality [25]. In a survey of hospitalized patients, noises and lights were identified as the main factors contributing to sleep disturbances, although it should be noted that patient perceptions may differ from objective data due to irritation bias [29]. This bias results in patients rating staff conversation as more disruptive than alarm noises despite polysomnography showing otherwise [20,21]. This does not mean that patient feedback has no clinical purpose, but it needs to be considered along with monitoring data.

With regards to noise in the hospital environment, research has shown that most ICUs operate at sound levels between 55 and 60 dB, far exceeding the WHO-recommended limits of 45 dB during the day and 35 dB at night [30]. Peak sound levels in these environments were recorded at 80 dB, which is correlated with a 35% chance of arousal in sleeping patients [31]. However, average sound levels are not the only benchmark for determining whether an environment is conducive to sleep. Sound peaks, which are sudden elevations in sound levels of more than 10 dB, have also been shown to disrupt sleep. In a study by Gabor et al., arousals from sleep were measured in control and in noise-reduced ICU rooms. Despite effective noise attenuation in the noise-reduced room, the frequency of arousals remained consistent for both groups, presumably due to sound peaks occurring in both. When the sound peaks were removed, arousal indices were significantly reduced [31]. A final feature of sound that has been found to play a role in sleep disturbance is the source of the sound. Research has shown that electronic alarms used to alert staff of changes in patient conditions have a greater impact on sleep disturbances than human voices [30,32]. This may be due to the prevalence of alarms in hospital environments and the relatively close proximity most monitoring equipment has to a patient’s head. These different aspects of sound all contribute to decreased sleep in the hospital environment and should be limited to improve patient sleep quality.

In addition to the noise levels, staff activities have also been linked to increased sleep disruption in hospital environments. Studies have shown that up to 7% of sleep disruptions in hospitalized patients are related to staffing-related care interventions [19]. While not ideal, many staff activities are necessary and ultimately benefit the patient, such as routine awakenings in neuro-intensive patients that are performed to screen for cognitive deterioration [33]. Often, these checkups occur with the highest frequency upon initial admission to the ICU and then decrease in frequency once patient stabilization occurs [28].

Proper staffing also requires adequate lighting which can further exacerbate sleep disruptions. Light plays an important role in the control of the circadian rhythm by inhibiting melatonin secretion from the pineal gland and delaying sleep onset. When patients experience increased light levels during the night, it disrupts their circadian rhythm and results in daytime drowsiness and nighttime insomnia [16]. This effect is compounded by inadequate light levels during the day, which produce a dim environment that can further disrupt the circadian rhythm and nighttime sleep [19]. Despite these negative effects, staff activity has also been shown to play a positive role in sleep. When surveyed, a significant number of patients reported feeling more comfortable and less anxious knowing that there was a strong staffing presence available to them, which, in turn, helped them to relax [34].

### 2.3. Surgical Factors

Various surgical parameters have been associated with increased rates of PSD, including the method of anesthetization used. While an anesthetized patient may appear to be asleep, anesthesia produces a state that is more akin to dreamless sleep or a comatose state [35] and has varying effects on sleep based on the agent used. Patients sedated using isoflurane have been reported to experience an increase in NREM and slow wave sleep immediately post op compared to controls [36]. Desflurane, on the other hand, might decrease slow wave sleep for up to 2 days following surgery [12]. However, determining the effects of each anesthetic agent has proven to be challenging. One study reported that, when comparing desflurane to propofol cohorts, patients reported similar sleep quality on the PSQI despite having significant differences in the levels of REM, NREM, and wake-after-sleep onset [12]. This inconsistency between different measurements makes it difficult to differentiate the effects of each anesthetic agent.

The route of administration may also play a role in the effect anesthesia has on PSDs. A systemic review observed that patients who were anesthetized using IV propofol had superior sleep quality compared patients receiving inhalational anesthesia [37]. Another study conducted on women following hysterectomies saw a significant improvement in postoperative sleep quality if the surgery was performed under spinal anesthesia instead of general anesthesia. This was believed to be due to reduced postoperative opioid consumption in the patients who received spinal anesthesia [38].

One potential underlying factor of anesthesia’s role in sleep disturbances is altered melatonin secretion. Anesthesia, in conjunction with surgery, causes a delay in melatonin secretion, resulting in melatonin levels plummeting during surgery and then rebounding to above average levels afterwards [35,39,40]. While increased melatonin levels may seem beneficial to sleep quality, studies have shown that this causes a shift in the cyclic secretion of melatonin and, instead, produces higher daytime levels of melatonin. This works to increase daytime sleepiness and causes more sleep–wake disturbances at night. These effects were observed in both sevoflurane- and desflurane-based anesthetics when assessed using rat models [41].

The timing and type of surgery have also been observed to play a role in developing PSD [42]. Studies have shown that sleep quality in young adults and middle-aged patients was heavily influenced by the surgical start time. Patients with surgeries that occurred in the afternoon (14:00–18:00) had increased levels of cortisol and decreased melatonin on postoperative night one than patients who underwent surgery in the morning, resulting in the afternoon group having almost twice the rate of sleep disturbances (70% of patients vs. 39%, respectively). It is believed that patients with morning surgeries may achieve better sleep due to having a recovery period that does not overlap with a sleeping period [43]. Interestingly, these results were not observed in older patients, presumably due to their decreased metabolism, which requires longer periods of time to recover from surgery [42]. With regards to how the type of surgery affects the development of PSDs, major surgery has been found to have a greater suppressive effect on slow wave sleep and REM sleep than minor surgery [7]. Gögenur et al. found that sleep quality after laparoscopic surgery returned to normal values in under a week compared to major abdominal surgery sleep recovery, which took over 4 weeks. This is believed to be related to the increased levels of trauma that occur in major abdominal surgery. Additionally, major abdominal surgery usually necessitates longer hospital stays, while laparoscopic patients may be discharged from the hospital on the same day. This allows them to sleep in their own homes sooner than major abdominal surgery patients, which may contribute to more rapid sleep recovery [44].

### 2.4. Physiological Response

In contrast to the external factors of the hospital environment and surgical parameters, PSD is also controlled by internal physiological responses to surgery. One such factor is inflammation, which may be more important than the surgery itself. In a study comparing the sleep quality of patients undergoing cardiac surgery for pericarditis, it was determined that there was no clear correlation between the type of cardiac surgery, type of heart failure, or type of valve involvement in predicting sleep quality. Rather, the major predictive factor was duration of pericarditis condition. In these patients, levels of inflammatory biomarkers (preoperative ESR, postoperative CRP levels, and postoperative IL-6 levels) were negatively correlated with sleep quality [45].

Another factor that is closely related to inflammation is pain. Pain has a bidirectional relationship with sleep in that increased pain causes decreased sleep quality and lack of sleep heightens the perception of pain [4,14,46,47]. Higher pain levels are associated with decreased REM and slow wave sleep, which may last months after a surgery [2]. On top of that, research has shown that patients who retire to bed for more than 2 h when attempting to cope with pain are more likely to experience pain chronification. Behavioral theory suggests that lying in bed while in pain creates a conditioned association between the stimuli, delaying sleep onset due to the previously experienced pain in bed [48]. As a result, pain is cited as the most common cause of PSDs [7].

### 2.5. Pharmacological Factors

Unfortunately, several of the analgesics that are used to attenuate post-surgical pain have also been associated with disrupted postoperative sleep structure. Sedatives such as benzodiazepines have been shown to reduce pharyngeal muscular support and tonicity. This can lead to airway occlusion when asleep and cause sleep fragmentation, something that is especially problematic in patients who already struggle with obstructive sleep apnea (OSA) or obesity [49].

Another class of analgesics that can cause sleep disturbances are opioids. Research has shown that accessory inspiratory muscles are paralyzed during REM sleep, which predisposes patients to respiratory failure during this stage. To protect against this, patients who are experiencing respiratory failure tend to decrease the amount of REM sleep they experience [50]. This same protective mechanism is also thought to occur in patients on opioids, as opioids can mute the body’s respiratory drive and cause hypoventilation and hypoxia. By reducing the levels of REM sleep, the body can avoid transient paralysis and prevent further exacerbation of opioid-induced hypoxia [24]. Opioids also pose a challenge because they may cause altered responses depending on a patient’s genetics. Recent research has shown that patients who are homozygous with the *GG* allele of the *OPRM1* gene experience increased sleep disruption when using opioids compared to patients with a different genetic composition. This is believed to be due to altered responses to opioids within the pontine reticular formation of the brain [51].

Lastly, NSAIDS have also been investigated for their role in producing PSDs. Prostaglandins, the major target of NSAIDs, serve an important role in regulating melatonin secretion and body temperature. When patients were administered a single dose of aspirin or ibuprofen, researchers observed an associated decrease in body temperature drops and melatonin secretion [52]. However, despite their effect on sleep-related processes, NSAIDs may not cause large effects on sleep quality. One study was unable to detect sleep structure changes in patients who were given ibuprofen when assessed using either polysomnography or subjective assessments [53].

## 3. Effects of PSD

### 3.1. General Implications of Sleep Loss

Loss of sleep results in both short- and long-term harmful effects, regardless of what the cause may be. Acute sleep deprivation has been found to induce the epigenetic remodeling of circadian clock genes, which can produce a feedback loop where the disrupted circadian rhythm increases sleep loss and sleep loss works to further disrupt the circadian rhythm [54]. This sleep loss can result in multiple mental disturbances including visual and hearing impairments and decreased cognitive performance [55,56]. As little as 6 h of sleep loss has been found to lead to increased false memory formation, misinformation, and impairments within the hippocampal complex [57,58]. Emotionally, sleep loss is associated with increased rates of depression and anxiety [59]. Outside of these cognitive processes, sleep loss also affects bodily functions and signaling. Less than one week of sleep loss in healthy young people was associated with dramatic changes in metabolic and endocrine function [60]. Specifically, sleep loss has been found to increase rates of obesity by raising levels of ghrelin secretion (an appetite-inducing hormone) and decreasing levels of leptin secretion (an appetite-suppressing hormone) [61]. Long-term lack of sleep is also associated with increased inflammation, higher rates of cancer, and accelerated tumor progression due to elevated levels of proinflammatory cytokines such as Interleukins 1 and 6 (IL-1, IL-6) and Tumor Necrosis Factor (TNF) [62].

### 3.2. Cognitive Impairment/Delirium

When focusing specifically on postoperative sleep loss, the significant degradation of cognitive function is a well-known side effect. These symptoms can vary widely but may present as impaired memory, poorer decision making, reduced emotional processing, impaired motor function, or decreased alertness [63]. This has been shown to result in significantly decreased performance on Sternberg (memory) tests postoperatively [64].

PSD has been found to change the number and function of synapses, leading to decreased white matter volume and stimulating the abnormal activity of the Hypothalamic–Pituitary axis, affecting cortisol secretion and cognitive function [65]. This leads to impaired memory and spatial cognition, ultimately leading to postoperative delirium (POD), one of the more significant and concerning symptoms of PSD [65,66]. POD is the severe impairment of mental cognition and performance, commonly characterized by cases of disorganized thinking, lapses in concentration, and the fluctuating disturbance of mental status [67]. POD is a frequent complication in ICU patients, occurring in 70–80% of patients [68,69,70]. POD is believed to be partially caused by the flattening of melatonin production at night and cortisol fluctuations throughout the day that occur in patients with PSD [30]. POD is a major concern in hospital settings because it has been found to be a predictor of higher mortality rates and longer hospital stays. In mechanically ventilated patients, those who developed POD had a mortality rate of 34% compared to the 15% mortality rate of those who did not develop POD [71]. It has also been shown that the development of POD was the strongest predictor of length of stay in hospital in non-ventilated patients, even after adjusting for severity of illness and other covariates [70,72]. POD also usually leads to worsening mental and physical status even after discharge, and most patients who develop POD have persistence of delirium beyond ICU stay [68,69]. This results in decreased recovery and poorer prognosis following surgery.

### 3.3. Metabolic Effects

Sleep curtailment due to PSD has also been shown to impair glucose tolerance and enhance the dysregulation of insulin, which can intensify inner physiological stress and cause insulin resistance [17,60,73,74]. This effect appears to be related to tissue-specific alterations of genome-wide DNA methylation states, leading to the hypermethylation of important clock genes. Specifically, research has shown that the sleep-loss-induced hypermethylation of the clock genes *Period Circadian Protein Homolog 1 (PER1)* and *Cryptochrome 1 (CHRY1)* contributes to glucose intolerance [73].

The development of glucose intolerance and insulin resistance can lead to many metabolic complications and chronic issues. Decreased carbohydrate tolerance is a well-recognized risk factor for the development of obesity, hypertension, and diabetes and can impair many important functions of the immune system [75]. The metabolic and endocrine alterations seen during the sleep debt condition, therefore, mimic some of the hallmarks of aging, which suggests that chronic sleep loss could increase the severity of age-related pathologies, such as diabetes and hypertension [60]. Insulin resistance was also associated with a significantly longer ICU stay (8 days vs. 4.5 days) in brain trauma patients who required surgery [76].

### 3.4. Stress Implications

Higher cortisol levels (the primary stress hormone) are often observed in individuals that are sleep deprived and suffering from PSD in hospital settings [77,78,79]. Normally, healthy amounts of sleep can suppress the Hypothalamic–Pituitary–Adrenal (HPA) axis, which controls the release of cortisol. However, in sleep-deprived individuals, the deep sleep that is required to inhibit the HPA axis is missing, which causes the over-activation of the HPA axis and leads to higher levels and the reduced regulation of cortisol [80,81].

Increased cortisol levels have many negative effects on recovery from surgery and length of hospital stay [82]. The oversecretion of cortisol from the HPA axis leads to inhibition of pro-inflammatory cytokines such as IL-1 and IL-6, which decreases immunoregulation and inflammatory responses, increasing susceptibility to post-surgical infections [2]. Additionally, elevated cortisol levels have been shown to lead to many chronic diseases including autoimmune diseases, asthma, rheumatoid arthritis, and chronic fatigue and have also been associated in age-related insulin resistance and memory impairments [60,83]. In the postoperative setting, elevated cortisol levels have been linked to delayed wound healing, increased length of stay in the hospital, and increased mortality [82,84]. It is important to note that, while higher levels of cortisol are not directly linked to mortality rates, cortisol along with other biomarkers (like Body Mass Index [BMI]) can be used as indicators of mortality [85].

### 3.5. Cardiovascular Implications

Chronic sleep loss in PSD has been demonstrated to increase the rate of cardiovascular events and aid in the development or worsening of cerebrovascular diseases, hypertension, and cardiovascular diseases [86,87,88]. Studies have shown that PSD can cause increased risk of strokes and lead to the development of sleep disordered breathing habits, which can cause obstructive sleep apnea (OSA) and further increase a patient’s risk of suffering a stroke [86,87]. Sleep curtailment has also been linked to hypertension, with estimates showing that each hour of sleep loss is associated with a 37% increase in the risk of becoming hypertensive [89].

The development of OSA from PSD has been found to be an independent risk factor for readmission within 30 days of discharge, with readmission rate of patients with OSA being 11.4% vs. 7.4% in patients without OSA [90]. Hypertension was also presented as an independent risk factor for length of stay in hospital, and patients with hypertension had statistically longer lengths of stay [91].

### 3.6. Pain Perception

As previously mentioned, PSDs have a unique bi-directional relationship with pain perception. However, the effect that poor sleep quality has on pain perception is larger than the effect pain has on decreasing sleep quality [92,93], and just one night of sleep deprivation is enough to aid in the development of hyperalgesia [94]. Opioid receptors found throughout the nervous system dull pain perception and increase descending pain inhibitory pathways, but sleep deprivation deteriorates the endogenous opioid system’s ability to decrease pain perception [2]. Along with changes in the opioid system, decreased sleep has also been linked to increased levels of monoamines and adenosine, which may play a role in increasing pain perception [2]. Physical effects can arise from this pain as PSDs have been associated with functional limitations at the 3-month timepoint in patients who underwent total knee arthroplasties, presumably due to increased pain perception that contributed to reduced mobility in the joint following surgery [95]. To complicate matters, however, not all consequences of sleep deprivation increase pain perception. Increased levels of nitrous oxide and orexin that occur with sleep deprivation have been shown to provide analgesic effects and increased levels of cortisol works to decrease inflammation, which, in turn, can decrease pain perception [2]. This complex interplay between pain and sleep makes pain a challenging yet important consequence of PSDs.

### 3.7. Delayed Recovery

**Figure 2:**
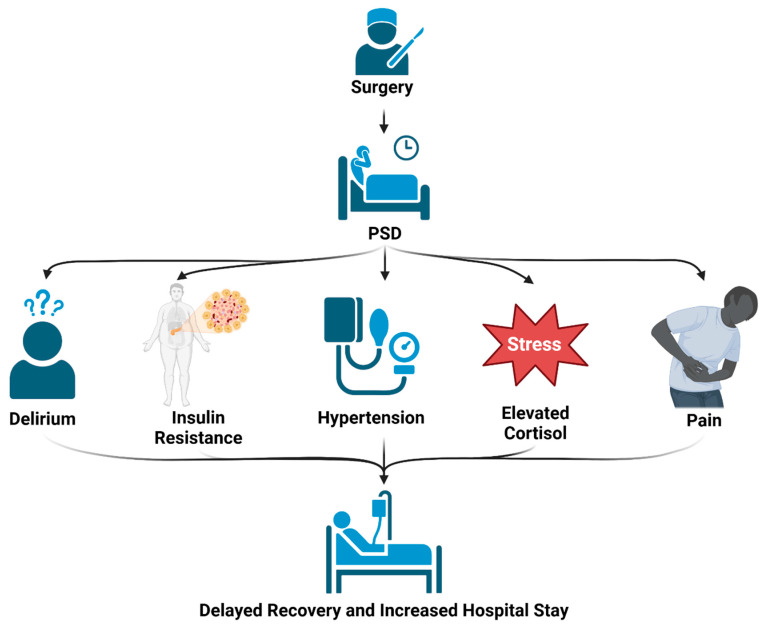
Effects of PSD: Sleep disorders that occur following surgery can lead to multiple complications, including confusion, metabolic dysregulation, hypertension, increased cortisol levels, and heightened pain perception. These detrimental effects can all contribute to increased length of hospital stay and poorer recovery.

As shown in previous sections, one of the major consequences of PSD is delayed recovery and longer stays in hospitals, which emphasizes the importance of good quality sleep postoperatively. One study reported that sleep disturbances are a significant predictor of mortality within one year of discharge among older people [96]. It has also been shown that quality of sleep in the first night post operation is an important factor in determining length of stay in hospital, even on fast-track recovery. Patients who had good quality of sleep were seen to have shorter hospital stays (42 ± 16 h) than those with bad quality of sleep (52 ± 10 h) [38]. Sleep disturbances have also been found to disrupt immune function by decreasing the number of lymphocytes (predominantly granulocytes and monocytes) excessively each day you go without sleep, which could lead to delayed recovery and longer hospital stay [97]. Metabolic syndromes that can develop from PSD have been correlated with prolonged length of stay in hospital and possible admittance to another hospital instead of being discharged [98,99]. A summary of some of the major factors that lead to decreased postoperative recovery can be found in Figure 2.

## 4. Treatments for PSD

**Figure 3:**
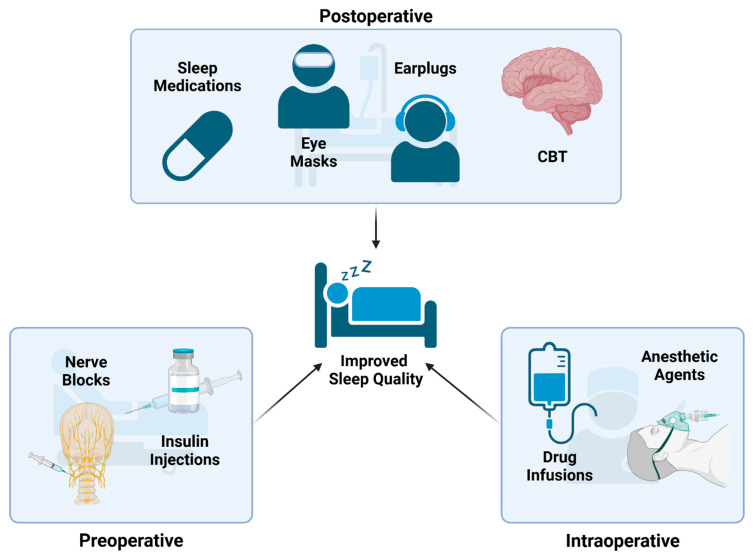
Treatment Strategies for PSD: These are several examples of treatment strategies that can be used at different timepoints during the operative process to reduce the occurrence of PSD. Preoperative interventions include nerve blocks and insulin injections. Intraoperative interventions include tailoring anesthetic agents and intraoperative infusions. Postoperative interventions include providing melatonin/other pharmaceuticals, eye masks, earplugs, noise masking, and cognitive (CBT) therapies.

Given the wide range of causes and long-lasting effects associated with PSDs, a large amount of research efforts have been devoted to treating them. The good news is that many of the conditions from which they arise have targetable interventions [4]. The enhanced recovery after surgery protocol (ERAS) outlines an approach to perioperative care that focuses on providing the best recovery outcomes to patients after surgery. This includes multiple steps that may help improve patient sleep, such as providing preoperative information to reduce anxiety, using minimally invasive surgery techniques, and employing advanced pain management techniques [7]. In addition to these procedures, many interventions throughout the perioperative timeframe are being investigated for their role in reducing PSDs (see Figure 3 for an outline of some of the areas of this research).

### 4.1. Preoperative Interventions

Researchers are investigating ways to decrease the risk of PSD at every step of the surgical process, including in the preoperative environment [4]. A recent study found that delivering insulin to patients intranasally starting 2 days prior to surgery reduces the incidence of postoperative delirium and improves sleep quality for 3 days following surgery. This is thought to occur through the regulatory effects insulin has on the HPA axis that reduce cortisol release and improve the endocrine system’s ability to regulate sleep [65]. Additionally, something as simple as screening for OSA prior to surgery and adjusting anesthetic techniques based on these findings has been found to improve recovery and sleep quality postoperatively [4]. Another method that has been investigated for reducing PSD is using local anesthetics to block the stellate ganglia, a sympathetic nerve bundle located in the neck, prior to surgery. This has been found to improve sleep efficiency, sleep maintenance, and total sleep time for the first two days following gastric surgery. This was also associated with reduced inflammation, increased melatonin levels, and attenuated sympathetic stimulation, which further contributed to improved sleep quality in these patients [100].

### 4.2. Intraoperative Interventions

The intraoperative period is another key point that offers many opportunities to reduce the risk of developing PSDs, with one such factor being the anesthetic agent used. In a study comparing desflurane and sevoflurane, it was found that both resulted in similar postoperative melatonin levels despite sevoflurane patients experiencing slightly less postoperative fatigue. This was surprising considering the faster recovery time of desflurane, but it led researchers to conclude that desflurane was equivalent to sevoflurane regarding PSDs [16]. When comparing propofol to desflurane in breast cancer surgery, it was discovered that both had similar overall effects on reported sleep quality for up to 30 days post operation. However, there was a difference in sleep structure, with propofol having a larger impact on REM and wake-after-sleep onset times, while desflurane increased NREM sleep [12]. From a more generalized standpoint, research has shown that combinations of generalized and regional anesthesia may have a superior effect on postoperative sleep quality compared to general anesthesia alone [37]. Based on these studies it seems that the choice of anesthetics may play a role in the development of PSDs; however, conclusive results have proven difficult to obtain. This may be due to the complexity of anesthesia and variations in drug metabolism from patient to patient that confound the data. As a result, further research is needed in this area to elucidate different anesthetic agents’ ability to reduce PSDs.

Aside from major anesthetics, other agents that can be delivered as intraoperative infusions are being investigated for their role in reducing the incidence of PSDs. Magnesium sulfate is one such agent, thanks to its potential ability to regulate melatonin production. When given to patients undergoing lumbar fixation it was found to significantly reduce both postoperative insomnia and pain [22]. Esketamine is another drug that is growing in popularity partially due to its ability to improve sleep-related symptoms of major depressive disorder, such as insomnia and reduced sleep quality. Researchers found that giving esketamine intraoperatively significantly reduced the incidence of PSD and decreased opioid consumption in the 24 h following surgery, presumably by reducing inflammation and helping to regulate circadian rhythms [18]. In another study by Cui et al., patients undergoing laparoscopic gynecological surgery were given intraoperative infusions of sodium oxybate, a partial GABA agonist that has been shown to induce slow wave sleep and REM sleep. Patients who received the drug reported significantly improved sleep quality on postoperative days one and three [14].

While these are promising results, not all drugs have shared this success. When given intraoperatively, dexmedetomidine (an alpha-2 receptor agonist) was not found to significantly improve reported incidence of PSDs despite producing a decrease in nocturnal wakefulness when used with sevoflurane and an increase in deep sleep when used with propofol [9]. Intraoperative lidocaine infusions also failed to significantly affect sleep quality on postoperative days 7 and 30 in patients following thyroidectomies. It should be noted that researchers in this study also failed to observe a decrease in the sleep quality of the control patients, which may be due to the relatively mild nature of thyroid surgeries [101]. Perhaps testing lidocaine in more intense operations will reveal a significant difference. Regardless, further research should be conducted to verify the effects each of these drugs have on the development of PSDs and to ensure their appropriate administration.

### 4.3. Postoperative Interventions

Of the three main timepoints to intervene in the development of PSD, the postoperative period is perhaps the most well studied. Current research has focused on both pharmacological and non-pharmacological methods of controlling PSDs and data suggest that both can have a positive effect on improving sleep quality.

#### 4.3.1. Postoperative Interventions—Pharmacological

Pharmacological approaches have long been the focus of attention for treatment in the postoperative timeframe [47]. Drugs that have classically been used include opioids and sedatives such as benzodiazepines and “Z-drugs”; however, these have fallen under scrutiny due to their potentially harmful side effects [2]. Along with decreasing pain, opioids might also reduce a patient’s respiratory drive and bind opioid receptors that are involved with sleep, which can lead to nocturnal hypoxia and sleep fragmentation [4,37]. Benzodiazepines and related drugs induce sleep but may also cause delirium and confusion [4]. To avoid these complications, researchers have been looking for alternative sleep-inducing drugs such as melatonin receptor agonists, dexmedetomidine, antidepressants, gabapentinoids, and cannabinoids [3].

Perhaps the most well studied drug class in this group is melatonin and melatonin receptor agonists. Research has found that many patients experience a decrease in endogenous nighttime melatonin levels following surgery that is lowest on postoperative night 1 and gradually increases back to baseline over several days. This dip is thought to contribute to PSD since melatonin is one of the chief hormones responsible for regulating the sleep–wake cycle [40,44]. The direct connection melatonin has to sleep as well as its good side effect profile, relative safety, and availability as a low-cost over-the-counter medication made exogenous melatonin supplementation quite popular with researchers [102]. Exogenous melatonin administration in the perioperative period has been shown to improve sleep quality shortly after surgery in patients undergoing total hip arthroplasties [103], laparoscopic cholecystectomies [20], prostatectomies [104], and breast cancer surgeries [10]. It has also been linked to lower rates of postoperative delirium and shorter hospital stays for patients following open heart surgery [105].

Despite this, not all research supports the use of melatonin. A study by Kirksey et al. found that melatonin did not significantly improve sleep quality when administered to patients following a total knee arthroplasty [106]. In addition to this, there have been mixed results in the benefits of the long-term administration of melatonin following surgery. When looking at 6 weeks postoperatively, melatonin was observed to improve sleep quality after arthroscopic rotator cuff surgery [23] but did not provide a significant improvement in sleep after orthopedic trauma surgeries [13] or total knee arthroplasties [102]. A systemic review published in 2023 concluded that melatonin does not improve subjective sleep quality in patients following surgery [107]. This wide range of results may be due to variations between studies with regards to how melatonin was administered, the types of surgeries that were performed, and the timing of assessing sleep quality. To obtain more conclusive data, more standardized approaches should be used when determining melatonin’s effects on sleep quality.

In addition to melatonin, dexmedetomidine is another drug that is being studied for use in the postoperative setting due to its sedative and anxiolytic properties [37]. Dexmedetomidine works by agonizing alpha-2 adrenergic receptors, which, ultimately, increases GABA output from the brain, activating endogenous sleep-promoting pathways and increasing sleep efficiency [7]. This has been found to stimulate sleep patterns similar to N2 sleep more effectively than direct GABA agonists [108]. Research has found that dexmedetomidine infusions following orthopedic, cesarian section, and abdominal surgeries improved sleep quality by increasing N2 and total sleep time while decreasing N1 sleep [37]. It also appears that the route of dexmedetomidine administration may play a role in effectiveness, as sleep scores were higher in patients who received IV infusions compared to intranasal or intratracheal administration [37]. Despite this success, studies have reported hypotension and bradycardia occurring as a result of its administration. One study investigated this further by assessing the effects of dexmedetomidine when used with oxycodone and found that high doses of dexmedetomidine increased the risk of hypotension, while low doses had a much lower risk of hypotension and provided the same sleep benefits [109]. More research is needed to determine the proper dosing of dexmedetomidine to reduce these negative side effect and maximize sleep potential, but initial testing appears promising.

Gabapentin is another drug that has been investigated for its effects on postoperative sleep. Its ability to block postoperative pain and increase sleepiness/sedation makes it a promising candidate to treat PSD [110]. Unfortunately, researchers did not detect a significant improvement in sleep quality after administering gabapentin to patients following orthopedic surgery [110]. NSAIDs have also been used to decrease pain and improve sleep in the postoperative period, but their potential drawbacks regarding hormone regulation that were mentioned previously leave the door open to new and more efficacious pharmacologic treatment options [2].

#### 4.3.2. Postoperative Interventions—Non-Pharmacological

While pharmacological interventions hold promise, they are also known to cause tolerance, dependance, rebound insomnia, and withdrawal. To help avoid these side effects, many non-pharmaceutical techniques are being investigated for their ability to reduce the occurrence of PSDs while offering lower risks, better prices, and increased convenience [111]. Recently, two systemic reviews were performed that assessed the effects of non-pharmacological interventions on sleep disturbances following cardiac surgery. These both concluded that interventions such as music therapy, exercise, cognitive behavioral therapy, massage, and acupuncture can significantly increase sleep outcomes in these patients [111,112].

One specific subset of these interventions relies on cognitive-based approaches [47]. In fact, the first-line therapy for general insomnia is cognitive behavioral therapy [2]. This technique focuses on educating patients about good sleep hygiene, restricts time in bed, and introduces patients to relaxation tools with the goal of improving sleep quality by implementing good sleep practices. This not only helps reduce sleeping disorders, but has also been found to reduce inflammation in adults [2]. In addition to changing patient behavior, another treatment option is to change the behaviors of the staff. Patients should be in quiet, dark, and comfortable environments at night and bright spaces during the day to maximize their sleep quality [4,19]. Having staff turn down monitoring alarms and be more cognizant about noise and light levels is an important step in achieving this. One study on nursery infants found that turning off radios, covering windows, and changing staff and visitor behavior led to improvements in the sleep duration and weight gain of the newborns [30].

Outside of the cognitive-based approaches, physical interventions have also been implemented to reduce rates of PSD. Using eye masks and earplugs is one of the most commonly studied methods since they offer a relatively inexpensive, well-tolerated, and noninvasive method to intervene with two of the major culprits of sleep loss: lights and noises [113]. Despite the amount of research put into this area, a consensus on their effectiveness at improving sleep quality has yet to be reached. A meta-analysis conducted in 2015 concluded that most studies did not show a benefit of using earplugs and eye masks [114]; however, studies that have been conducted since then have had mixed responses. One study that focused on patients following abdominal surgery found no significant improvement in sleep quality [28], while a different study focusing on ICU patients after breast surgery claimed eye masks and earplugs did significantly improve sleep quality [113]. A third rendition of this study determined that eye masks and earplugs increased sleep quality and decreased postoperative delirium in patients who had undergone a coronary artery bypass graft [115]. This disagreement amongst studies may be a result of varying patient compliance, with two studies reporting 35–40% noncompliance rates amongst patients who were supposed to be wearing eye masks and ear plugs [28,113]. This seems to show that, while these two interventions can successfully reduce noise and light issues, they are not a suitable solution to PSDs if patients cannot tolerate them.

Another intervention to block hospital noises is sound masking. This method uses white noise machines or ocean sounds to provide consistent background auditory stimulus and has been found to provide a greater improvement in sleep quality than earplugs, behavior modification, or acoustic absorption [30]. One study specifically found that having patients listen to Chinese five-element traditional music in the perioperative setting significantly improved sleep quality, decreased rates of delirium, and increased salivary melatonin levels on postoperative night one [116]. 

Acupoint therapy (acupuncture, acupressure, and electroacupuncture) has also been shown to positively affect postoperative sleep. These treatments originated in ancient China and are believed to work by changing the local activity of the brain and inhibiting the CNS to achieve deep sedation [117]. Studies have shown that these treatments can improve anxiety and depression pre- and postoperatively and reduce postoperative pain and inflammation [117]. Electroacupuncture in particular is believed to increase levels of serotonin and GABA, which can help to increase sleep quality [24]. There are several other related methods outside of these, such as aromatherapy, valerian acupressure, and massage; however, studies investigating these methods are lacking in both quality and quantity [114].

Aside from that, foot baths and transcranial direct current stimulation (tDCS) may also serve as beneficial postoperative interventions. Researchers have found that giving postsurgical patients 20 min foot baths in warm water or warm water with lavender oil prior to sleep decreases pain and increases quality of sleep, presumably by reducing pain and sympathetic activity [118,119,120,121]. Additionally, the weak polarizing currents administered during tDCS have also been found to increase deep sleep, REM, and total sleep on postoperative night one following lower limb arthroplasty if placed over the prefrontal cortex [122]. Lastly, health care providers should also make sure to treat conditions that contribute to decreased sleep quality, such as giving antitussives to patients with a cough [19].

### 4.4. Limitations on Data

While a large amount of data exists that focuses on reducing PSDs, we felt it necessary to address some of the potential limitations. One of the issues that we felt complicated data was lack of consistency between studies, particularly with how and when sleep quality was assessed. Of the studies we reviewed, the timing between surgery occurring and researchers assessing sleep quality varied from 1 day to 6 months postoperatively [23,110]. As discussed previously, the effect surgery has on sleep quality decreases the longer out you are from surgery and having this wide of a time range may have affected whether an intervention had a significant effect or not. Along with this, over nine different methods were used to analyze sleep quality across the papers we reviewed. This variation in data collection makes comparing studies quite difficult as each method has its own unique strengths and weaknesses. Additionally, these studies could be improved by conducting larger multi-center trials. Of the papers we reviewed, the study sized varied between 40 and 188 patients, and only one was listed as a multicenter trial [9,38,100]. Overall, we feel that creating standardized methods of assessing sleep quality and interventions will improve the data that exist and help provide better guidance for how to reduce PSDs. Further discussion on ways to improve the accuracy of data can be found in the following section.

## 5. Future Directions

One of the major areas for improvement within the field of PSDs is better sleep monitoring methods. Polysomnography is currently one of the best tools to gain objective data on sleep quality; however, its high price and relative complexity make it difficult for researchers to use. As a result, many of the studies in this paper utilize self-reported questionnaires as a quick and efficient alternative to obtain a base understanding of different treatment strategies. While this works, questionnaires have been found to be influenced by the context of their administration and patient psychological factors [13]. Additionally, the wide range of questionnaires that exist limits the ability for researchers to compare results between various studies [25]. Adding more objective measurements will add an extra angle of analysis to hopefully reduce the impact these variables have on studies and increase the reproducibility of the findings.

Recent developments in technology may offer new opportunities to gain objective data in increasingly efficient and ergonomic ways. One such improvement is new developments in fMRI, which allow researchers to measure EEG signals and CSF flow during sleep to better understand brain mechanics [6]. Bispectral Index (BIS) monitoring is another advancement that allows researchers to directly monitor a patient’s level of consciousness through EEGs [37]. However, these are limited by either functional capabilities or cost of operation that make them less ideal options for use in sleep research. 

Instead, other advanced forms of polysomnography may be the future. This includes respiratory polygraphy, which measures respiratory rate (RR), heart rate (HR), and SpO2 to assess the sleep quality of patients [123]. While limited in its diagnostic capabilities, the ease of administration and portability make this a potential alternative to classic polysomnography [123]. Another option is modular sleep monitoring devices. These have adjustable monitors that can be added or removed to fit the requirements of a specific study, allowing researchers to remove unnecessary monitoring devices while still offering the full capabilities of a traditional polysomnography device if needed [123]. Lastly, wireless polysomnography devices are being created to limit the number of wires that are attached to patients. These hope to offer improved comfort and ease of application over traditional polysomnography; however, factors such as data security, signal interference, and costs have limited their use [123]. For all these devices, advancements in technology are expected to improve their accuracy and reduce the cost, hopefully offering researchers better options to incorporate polysomnography into studies and improve sleep data. 

In addition to advancements in polysomnography, consumer sleep trackers may also serve a large role in the future of sleep research. Consumer sleep trackers are devices that offer a relatively cheap and affordable option for patients to monitor basic sleep variables and receive feedback on how to improve sleep habits [124]. Of these devices, most fall into one of three major categories: wearables, nearable, or airables. 

Wearables make up the largest area of this field and consist of devices that are worn by users, such as smart rings and smart watches [11]. These devices often monitor physiologic values through sensors like photoplethysmography, which utilizes light sources and photodetectors to determine HR, RR, and SpO2 levels. Photoplethysmography is often combined with actigraphy, which monitors movements while asleep to provide data on sleep stages, breathing habits, and sleep disorders [123,125]. Outside of watches and rings, other wearables include devices such as chest belts and head bands that can, respectively, monitor breathing patterns and EEG activity while asleep [123]. Of the wearable devices, EEG-based systems are considered the most accurate at detecting sleep stages, sleep latency, and sleep times due to their unique ability to directly detect brain activity [125]. Recent developments in technology and the general affordability of all wearable devices have made them a large focus of attention in sleep research. They are especially valuable in longitudinal studies with some researchers now using consumer-based wearable devices to collect sleep data, although studies have shown that these methods are still not as accurate as polysomnography [126].

In contrast to wearable devices, nearables and airables serve as contactless methods to monitor sleep. Nearables are devices that are placed near individuals or in furniture (e.g., mattresses) to detect movements and respiratory efforts throughout the night [11]. One example of these is ballistographic-based devices that are placed in the platform a patient is lying on to record HR, RR, and sleep movements. While not great at identifying sleep stages, they hold a lot of potential to measure sleep quality over long periods of time [123]. Other nearable devices use radar to detect sleep movement and respirations. While they are susceptible to external interference, these may offer researchers in the future a way to monitor sleep in patient populations who cannot wear other sleep devices, such as infants or the elderly [123]. Airables, on the other hand, utilize sound detection to monitor breathing patterns, snoring, and ambient noise levels. Many apps utilize this approach to help monitor sleep, making airables perhaps the most accessible form of sleep monitoring [11]. However, limitations in the data that can be collected and interference from external noise limit the use of ariables to mostly detecting sleep breathing disorders and sleep disruptions [123]. 

As technology improves, many of these consumer sleep trackers are becoming increasingly accurate, with some devices approaching the capabilities of polysomnography [123]. To validate this, a recent study by Lee et.al. looked to compare 11 different devices to polysomnographic monitoring. They found that an app utilizing airable technology excelled at identifying wakefulness and REM sleep stages, while wearables such as Google Pixel and FitBit watches were best at detecting the stages of deep sleep [11]. 

Despite the promising data that exist on these devices, a lack of validation studies has restricted them from being used widely in research. This is especially true with nearable and airable technology, which have much less validation studies than wearables [123]. As of 2025, the World Sleep Society advised that, while wearable consumer health trackers have many uses in healthy adults and people with sleep disorders, their performance must continue to be evaluated as technology is refined to fill in key gaps. Some of these key gaps include performance validation in younger children, older adults, and patients with chronic conditions [127]. Overall, for these devices to be widely incorporated into sleep research, further improvements must be made in data accuracy and more validation studies must be conducted across a wide range of patient populations. 

Outside of improvements in monitoring devices, further research into the intraoperative influences of PSDs would be beneficial to advancing the field. Specifically, we struggled to find data investigating the effects that more intense surgeries and trauma had on sleep disturbances and treatment strategies for these cases. Investigating these situations would provide valuable information to patients that may suffer the most from PSDs. Another complication is that many of the studies we reviewed focused on a specific subset of surgical patients. It would be beneficial to investigate the effects of some of the more well reviewed PSD therapies across a wide range of surgical and operative conditions, thereby increasing the generalizability of the findings and opening the treatment to a broader scope of patients.

In addition to expanding research conditions, increasing the number of studies that focus on intraoperative and preoperative interventions could be beneficial. Most of the data we found focused on postoperative interventions. A better understanding of the conditions that lead to PSDs may add prophylactic therapies that can be used in conjunction with postoperative interventions. Along with that, there is a lack of high-quality research into non-pharmacological methods of treating PSDs [114]. Improving this knowledge offers the opportunity of developing low-cost low-risk interventions to improve postoperative sleep quality.

## 6. Conclusions

PSDs are a serious medical challenge. They can arise due to influences at all points of the surgical process and result in poorer recovery and increased postoperative mortality. As a result, multiple treatment approaches are being investigated to reduce their prevalence. However, work still needs to be completed to optimize the administration and effectiveness of these techniques.
